# Basic and clinical study of the effect of exogenous hyaluronic acid on the quality of acellular dermal matrix combined with thin intermediate split thickness skin graft

**DOI:** 10.1186/s40001-023-01283-4

**Published:** 2023-11-06

**Authors:** Fuhuan Chen, Jiake Chai, Jingyu Zhao, Jiang Wu, Baoguo Chen

**Affiliations:** 1 Comprehensive ward, Plastic Surgery Hospital, Chinese Academy of Medical Sciences and Peking Union Medical College, Beijing, 100144 China; 2 Department of Burn and Plastic Surgery, Fourth Medical Center of Chinese, Burns Institute, Burn & Plastic Hospital of Chinese PLA General Hospital, PLA General Hospital, Beijing, 100048 China; 3 Medical Cosmetic Center, Beijing United Family Hospital and Clinics, 2 Jiangtai Road, Chaoyang District, Beijing, 100015 China

**Keywords:** ADM, Skin graft, HA, MVD, Biomechanical characteristics

## Abstract

**Background:**

To promote wound recovery in the recipient region, we studied the impact of exogenous hyaluronic acid (HA) on acellular dermal matrix (ADM) paired with thin intermediate-thickness skin transplant.

**Methods:**

This study contains animal and clinical experiments. 50 Japanese big ear rabbits were separated into HA1, HA2, PADM, TS, and NS groups. Clinical part included 50 scar patients dividing into 5 groups (TS + HA + ADM 1, TS + ADM2, TS, TS + ADM and normal skin (NS)).

**Results:**

In the animal trial, after 56 days, the grafts contracted least in the HA2 group; HA2 had the highest microvascular density (MVD), HA concentration, and collagen I and III expression. In clinical work, ADM > HA + ADM2 > HA + ADM1 > TS > NS; Type I and III collagen: HA + ADM1 and HA + ADM2 were higher than ADM; HA content: TS > HA + ADM1 > HA + ADM 2 > ADM.

**Conclusions:**

ADM, exogenous hyaluronic acid mixed with thin skin autograft has better biomechanical qualities and therapeutic impact than acellular dermal matrix alone, and the reconstructive result is near to self-thick skin autograft in all indexes.

**Supplementary Information:**

The online version contains supplementary material available at 10.1186/s40001-023-01283-4.

## Introduction

Skin grafts are more typically used to correct full-thickness skin abnormalities after burn contracture release, large tumor removal, breast cancer surgery, and other injuries [1–7]. Thick skin grafts are ideal for reconstructive surgeries because they carry more dermis and are less likely to produce scar recurrence. Secondary hypertrophic scar or delayed healing dissatisfy us when using thick or full-thickness skin transplants. The transmission of a composite skin graft can be accounted for by the non-immunogenic acellular dermal matrix (ADM), which has been devoid of dermal and epidermal cells. Since its initial release, ADM has been a significant supplement in the development of fibroblasts and blood vessels. It gives the neodermis a bridge and boosting qualities. As in contrast to previous studies, we have described this method of composite skin graft transfer as involving only one procedure. In our findings, thin intermediate split thickness skin transplant and ADM were used to achieve good outcomes in the recipient site and decreased morbidity and scarring in the donor site compared to standard skin graft [8].

**Fig.1:**
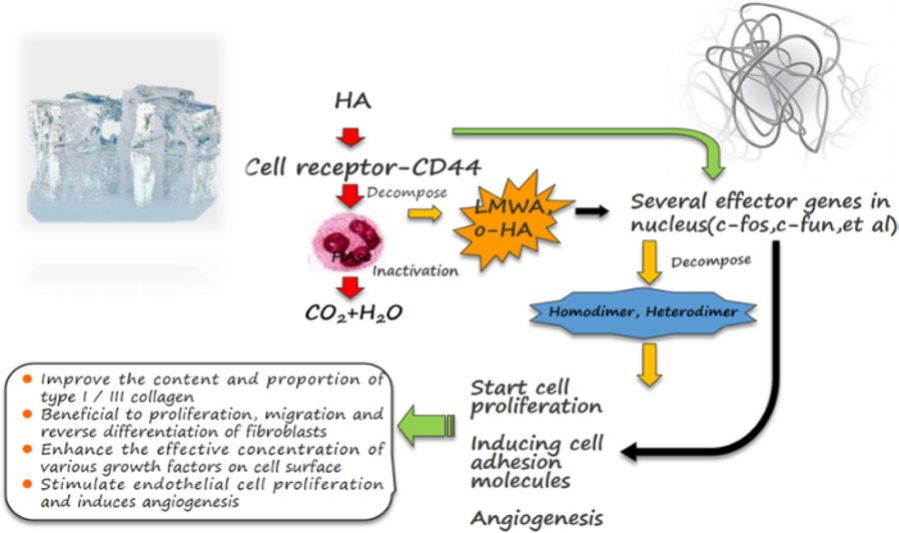
Biological role of hyaluronic acid (HA)

Even though ADM has been used clinically as a composite skin transfer for various types of reconstructions, despite hopeful results in the recipient and donor sites, composite skin grafts often constrict in the recipient site later. An evident reduction in hyaluronic acid is one of the potential explanations for these symptoms (HA). HA can act as an anti-inflammatory, controlling collagen synthesis and encouraging wound healing during the absence of scarring in fetal skin [9, 10]. During ADM decellularization, mucopolysaccharides in the extracellular matrix are always removed [11, 12] (Fig. 1). Therefore, the application of exogenous HA during ADM composite skin grafting is possible for regulating collagen synthesis and reducing scar development and graft contracture.

In this study, animal research and clinical research were conducted to determine whether exogenous HA can improve the quality of composite skin graft, changes in skin graft biomechanical qualities, graft contracture rate, collagen I and III synthesis, micro vessel density (MVD), and HA content.

## Animal research

### Methods

50 clean Japanese large-eared rabbits weighed 3.1 ± 0.1 kg. Four 5 cm × 3 cm rectangular skin sections were excised symmetrically from each rabbit's spinal column. Four centimeters separated the fragments. The wound extended to the fascia layer. 200 wounds were prepared. The experimental group’s full-thickness skin was reverse-sliced into 0.4-mm-thin slices of intermediate-thickness skin (TS) using a drum dermatome. Control group samples were normal skin (NS). A 1:3 ratio of xenogeneic porcine acellular dermal matrix (PADM) was generated using a sterile skin mesh puller. The PADM was cut into rectangles (7 meshes by 5 meshes). Five groups of 10 rabbits were split by treatment technique. They comprised HA1 + PADM + TS autograft (HA1), HA2 + PADM + TS autograft (HA2), PADM + TS autograft (PADM), TS autograft (TS), and normal skin (NS). For HA1, 0.3 ml of exogenous HA was administered to the skin graft's dermal surface (0.17 mg/cm^2^). The wound was covered with mesh-pulled PADM and HA graft (the dermal surface facing inwards). For HA2, methods were the same as for HA1, but the HA dosage was twice (0.6 ml, roughly 0.34 mg/cm^2^). PADM received no HA. Thin autografts were sutured in place for TS. All experimental wound surfaces were packed following suturing. For NS, postmortem back skin was obtained. Position, size, and texture direction were the same for all samples. All groups received rip-prevention and anti-infection therapy. Hospital ethics authorized the research. The specific surgical procedure is consistent with our previous publication [24].

The samples were taken at two distinct times. Ten rabbits were euthanized 28 and 56 days after operation. Gross samples were assessed for contracture rates, and grafted tissues were removed. The samples were split into two distinct groups. The liquid nitrogen was used to fast freeze one portion before crushing and lysing it. Collecting the supernatant fluid for Western blotting analysis. The remaining portion was kept in liquid nitrogen. Ten rabbits were euthanized on post-operative days 3, 7, and 14 during the second session. Four grafted tissues and the control skin were obtained from the rabbits' backs. Once again, the specimens were divided into two groups.

### Results

**Fig.2:**
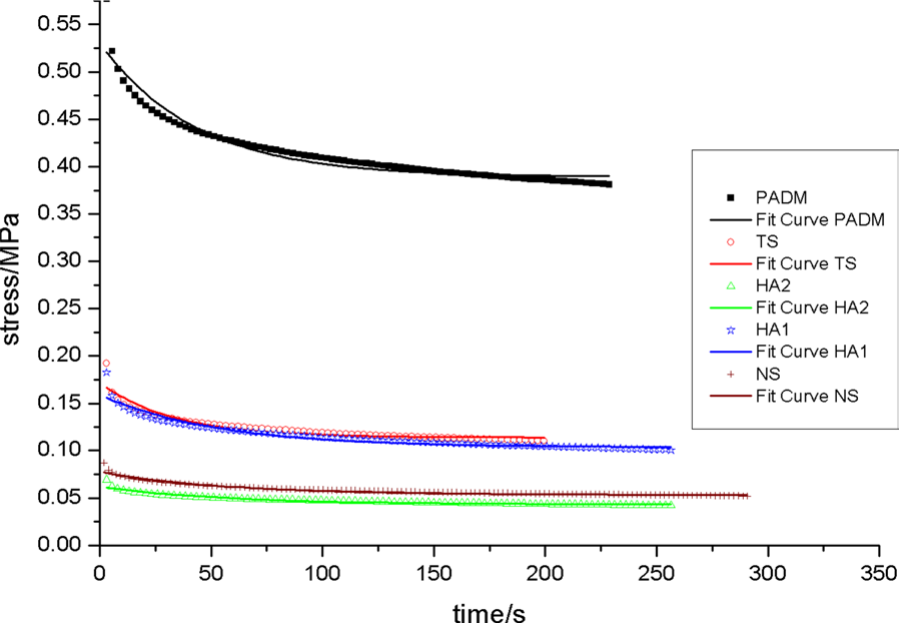
The relaxation curve of the 28d across the various groups

**Fig.3:**
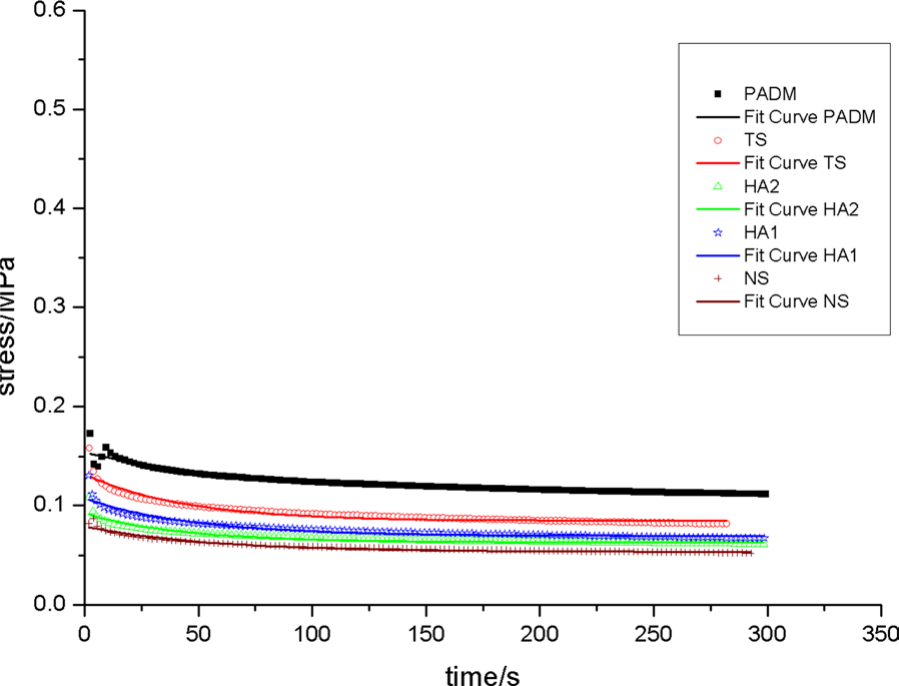
The relaxation curve of 56d for each of the various groups

**Fig.4:**
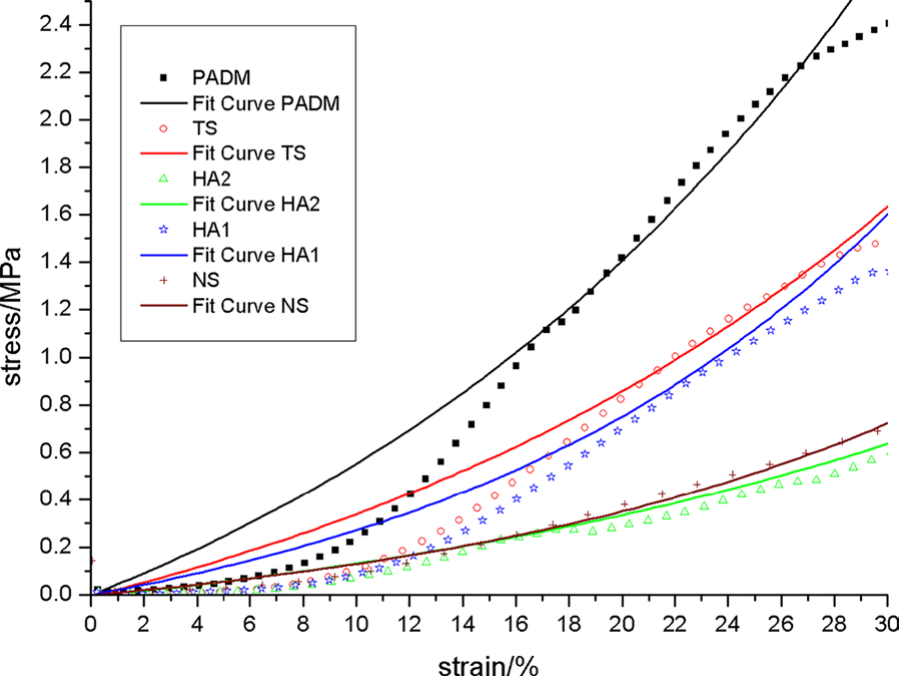
Stress–strain curves for different groups of 28d

**Fig.5:**
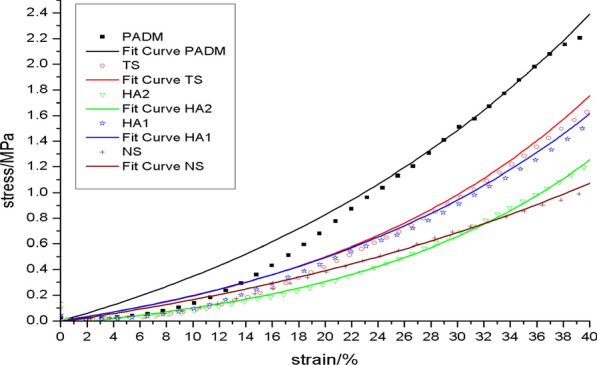
Stress–strain curve of 56d in different groups

1. The rate of graft contracture: On day 28 following surgery, the TS group had a greater graft contracture rate than the other groups. PADM, HA1, and HA2 did not vary significantly. On day 56 post-operatively, PADM and TS had higher contracture rates than other groups (*p* < 0.05), although HA2 had the lowest [24].
2. Biomechanical qualities: (1) the relaxation curve revealed that, on day 28 post-operation, the HA2 group’s curve was near to that of the NS group, as was the scenario with the HA1 group and the TS group (Fig. 2). On day 56 post-operation, the HA2 and HA1 groups’ curves were most like those of the NS (Fig. 3), while the PADM group's curve was most divergent from that of the NS.
3. On post-operative days 28 and 56, the HA2 curve was close the NS curve (Figs. 4 and 5). The flexibility of the skin in the HA2 group was superior to that of the other groups of grafts when subjected to the same level of stress, even though its strength was worse. While the curves of the PADM group differed significantly from those of the NS, and the PADM group was able to withstand excessive stress, they were less flexible than those of other groups.
4. Micro vessel density (MVD): On the third post-operative day, HA1 and HA2 had no capillary growth, but TS and PADM did. On day 7 post-operation, capillaries were detected in the HA1 and HA2 groups; there was no significant difference in capillary density between the HA1 and PADM groups; and the MVD in the SHA2 group was larger than that in the PADM and HA1 groups, but lower than that in the TS group. On day 14 post-operation, there was no significant difference in MVD between the PADM and TS groups, both of which were lower than the HA1 group, however the HA2 group had a higher MVD. On post-operative days 28 and 56, HA2 had the highest MVD, followed by HA1, TS, PADM, and NS. All experimental groups’ MVD values climbed 3–56 days post-operatively, then declined. [24]
5. Hyaluronic acid (HA) concentration: Both the HA1 and HA2 groups had higher HA concentrations than the PADM group on day 3 post-surgery. On day 7 post-op, no differences were identified between HA1, HA2, and PADM. On day 14 following surgery, HA1 and HA2 were both lower than PADM. On day 28 post-operation, there was no change between the HA1 and PADM groups, but the HA2 group had a decreased HA concentration. On day 56 post-operatively, there was no difference between the HA1 and PADM groups, while the HA2 group had a higher HA concentration [24].
6. Collagens (I and III) expression: On 28d after operation, type I and type III collagen in HA1 and HA2 groups was higher than that in PADM group, and type I and type III collagen in HA2 was higher than that in HA1. On 56d after operation, the difference was the same as in 28d group. Changes in type I and type III collagen composition in HA1, HA2, and PADM 28 and 56 days after surgery: type I collagen in SHA1 and HA2 reduced throughout time. In HA1 and PADM, type III collagen rose with time, whereas it decreased in HA2 [24].

## Clinical research

### Patients and methods

50 individuals between the ages of 5 and 50 who need scar excision and repair were identified. 16 patients with an upper extremity hypertrophic scar or scar contracture, 20 patients with a trunk hypertrophic scar or scar contracture, and 14 patients with a lower extremity hypertrophic scar or scar contracture exhibited scar affection. Diabetic, coagulation, and scar diathesis patients were excluded. Ten patients were randomly allocated to each of five groups: TS, TS + ADM, TS + HA + ADM1, TS + HA + ADM2, and NS (no treatment).

14 days following the procedure, the survival of the skin was examined. These patients were followed up on at 3 months and 1-year intervals. The following substances were identified: hyaluronic acid, collagen I and III expression, and biomechanical properties (measured by biotester biaxial tensile test machine).

The statistical program SPSS 20.0 was used for statistical processing. The People's Liberation Army General Hospital Committee accepted this research.

### Surgical technique

Each patient received one-stage surgery. The burned scar and contracture were removed under general anesthesia. In severe burns, careful resection is needed to protect deep tissue. We must preserve important structures for the following skin graft’s placement and coverage. After soft tissue release, the defect was filled with acellular dermal matrix utilizing absorbable sutures. Dermis matrix was submerged in saline water and washed twice to remove storage fluid. Thin split-thickness grafts were taken from the back, thigh, or scalp using a motorized dermatome (Zimmer, Illinois State, America). Each secondary defect was halved after excision or release. Part of the defect was HA’d. The remaining fault contrasted. The autograft was placed on the alloderm after HA was applied to ADM. The composite skin co-graft was packed to maintain pressure. Each packet was kept for 14 days. Seven-day intravenous antibiotics were given. After 14 days, the package was removed to test the graft. Vancouver Scar Score Scale was used to evaluate donor and recipient sites. All patients signed data-use consent forms.

### Results

The median age of patients was 26.6 (29 male patients and 21 female patients). The mobility function of all scarred hands was significantly enhanced in individuals with scar contracture. Infection, edema, and necrosis of the skin transplant were not seen in any of the patients. The whole skin transplant was successful.

*Aesthetic enhancement* In terms of flatness, pigmentation, and elasticity, the HA + ADM1 group was superior to the HA + ADM2 group, with no significant difference compared to the TS group, and considerably superior to the ADM group.

*The degree of donor site’s injury* In the TS group, the donor region healed in 14–16 days, leaving visible scars; in the HA + ADM1, HA + ADM2, and ADM groups, the donor area healed in 7–9 days, without visible scars after healing and leaving just superficial pigmentation.

*HA content of grafted skin* TS group > HA + ADM1 group > HA + ADM2 group > ADM group, the content decreased one year after surgery, the difference was significant (follow-up time: three months and one year after operation) [24].

*Expression of collagen I and III in grafted skin* Three months after the operation, the collagen of type I and type III in TS, HA + ADM1 group, and HA + ADM2 group was higher than that in ADM group; one year after the operation, the difference was the same as in the three months after the operation [24].

**Fig.6:**
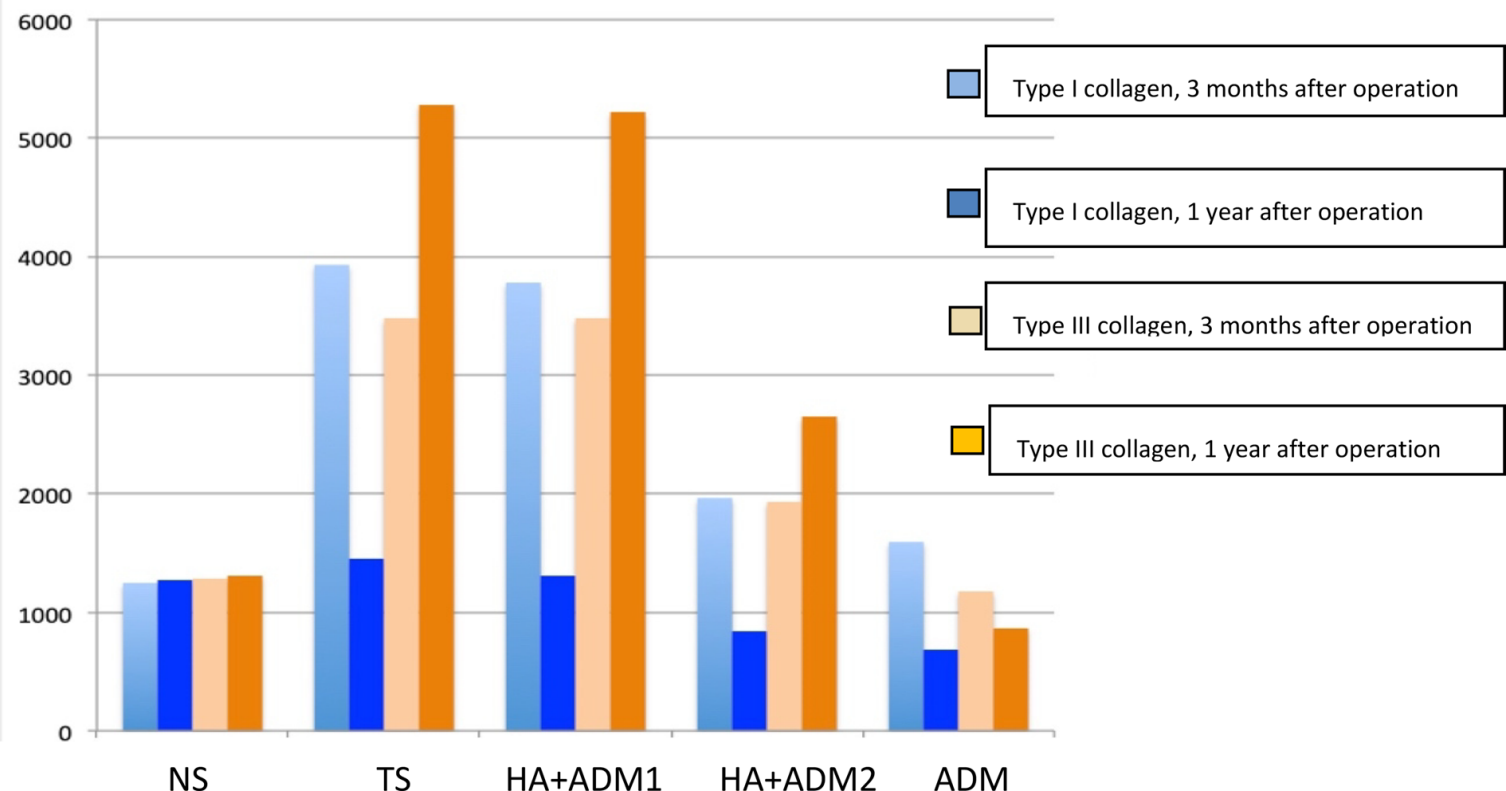
The average gray value of expression of types of collagens in each group (X ± S, *n* = 10)

*Change trend* (Fig. 6) type I collagen content of TS group, HA + ADM1 group and HA + ADM2 group decreased with time (*P* < 0.05), while that of ADM group increased gradually (*P* < 0.05); type III collagen content of TS group, HA + ADM1 group and HA + ADM2 group increased gradually with time (*P* < 0.05), but that of ADM group decreased gradually (*P* < 0.05).

**Fig.7:**
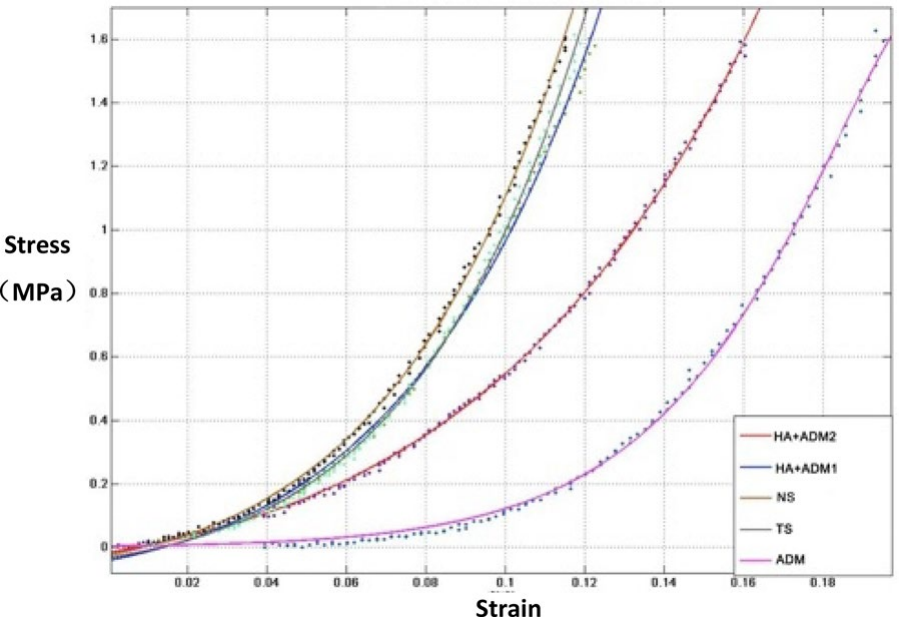
Stress–strain relation of different groups

Biomechanical parameters of the grafted skin: Under the same load, the deformation of each group is as follows: ADM group > HA + ADM 2 group > HA + ADM 1 group > TS group > NS group, with the elastic retraction ability of the normal skin being the best and ADM group being the poorest (Fig. 7).

### Case reports

Case 1 (Fig. 8).

**Fig.8:**
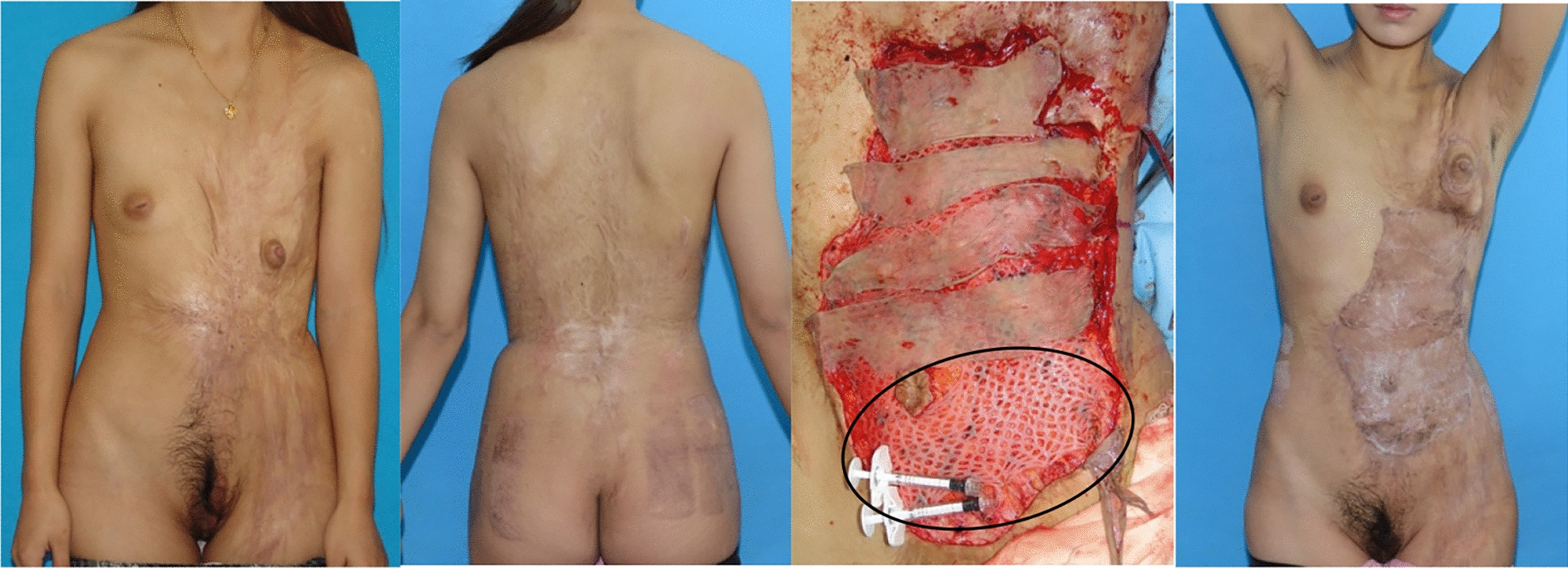
This patient suffered severe chest and abdominal burns. The bottom portion was fixed using auto-thin skin graft, ADM, and HA, while the top portion was repaired with auto-thin skin graft and ADM. After 1.5 years, the lower half of the body had healed more than the upper half. There is no evidence of hypertrophic scarring or delayed healing at the donor location

This patient suffered from burnt scar contracture of the chest and abdomen, which resulted in an undeveloped left breast and chest contracture belt with longitude. For reconstruction, we employed a thin skin graft from the back and hip region and autologous dermal matrix. HA was used in the bottom portion of the secondary defect outlined by the black line, as opposed to the higher portion. Despite the relaxation of scar contracture after one year, the underdeveloped breast was not symmetrical with the normal breast. There were no hypertrophic scars or delayed healing at the donor locations. The bottom portion of reconstructive tissue healed better with the addition of HA than the top portion did without the addition of HA.

**Fig.9:**
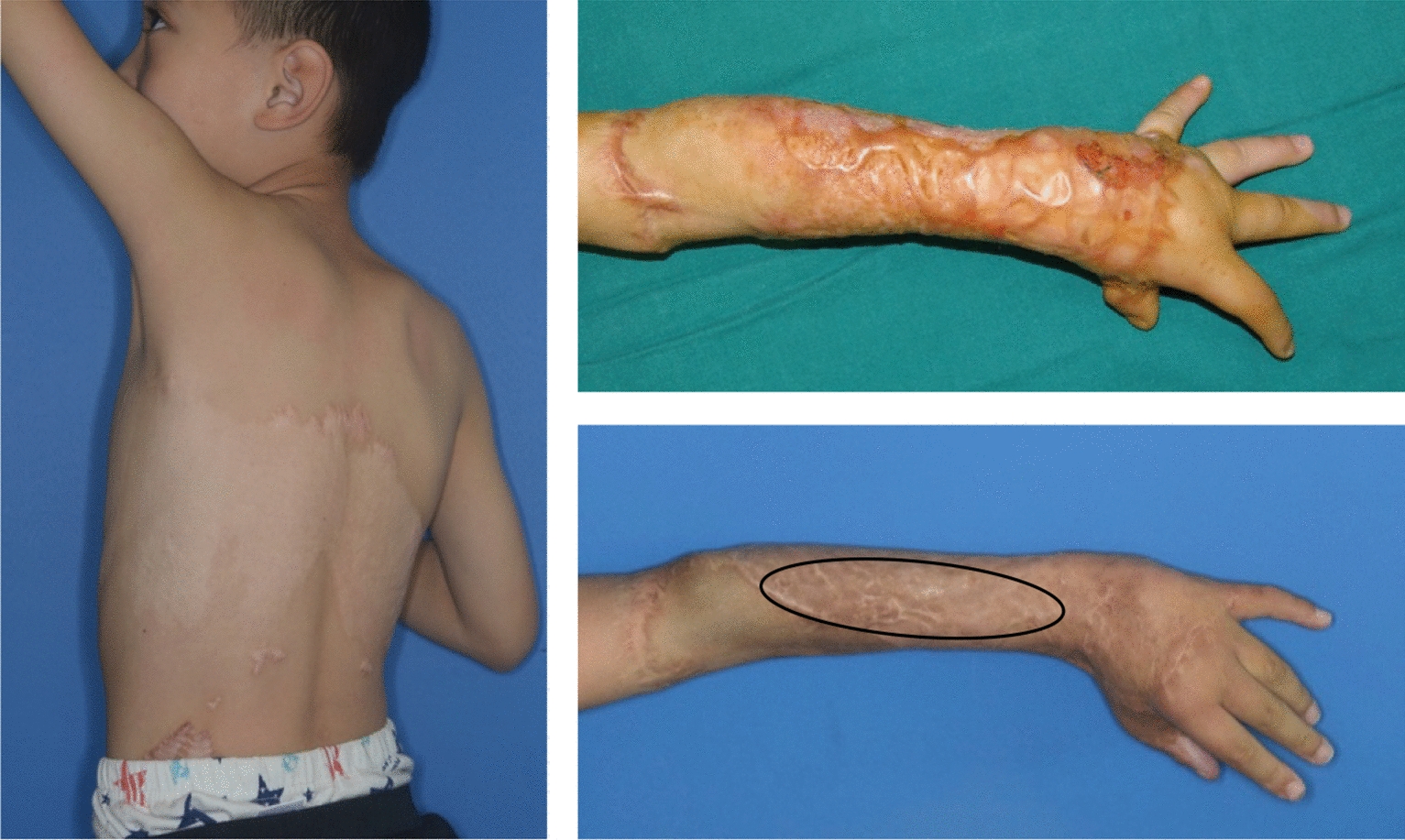
This young boy’s left upper arm has hypertrophic scarring and scar contracture. The region inside the black circle was healed using auto-thin graft, ADM, and HA, while the remaining area was fixed using auto-thin graft and ADM. The region that received HA recovered more quickly than the other location. The donor location on the back exhibited neither hypertrophic scarring nor delayed healing

Case 2 (Fig. 9/Additional file 1: Video S1).

The left upper extremities of this child were injured by scald. For two years, the hypertrophic scar inhibited movements. After removing the hypertrophic scar, the secondary defect indicated by the black circle was treated using auto-thin graft, ADM, and HA, while the other region was fixed using auto-thin graft, ADM. The auto-thin graft was transferred from the rear. After 40 months, the secondary region that was treated with HA healed better than the area that was not treated with HA. There is no evidence of hypertrophic scarring or delayed healing at the donor location.

## Discussion

Hyaluronic acid is a > 106 kDa polysaccharide chain made of glucuronic acid and acetylglucosamine. It is found in animal dermis and epidermis. Different tissues' HA concentration and molecular weight differed [13–16]. Wound healing involves angiogenesis and inflammation. Capillary development includes activation, migration, proliferation, and lumen formation. Due to its variable molecular weight, previous research has shown that the impact of HA in stimulating angiogenesis is vastly unique. High-molecular-weight HA may inhibit angiogenesis, whereas low-molecular-weight o-HA may promote it. Low molecular weight HA stimulates and increases endothelial cell growth. Slevin et al. showed that o-HA with low molecular weight may stimulate endothelial cell mitosis by interacting with CD44 and RHAMM and activating PKC and map signal transduction [17]. High-molecular-weight HA may impede endothelial cell migration to fissures [18]. Low molecular weight o-HA (between 600 and 2000) may increase type I and type VIII collagen, a crucial vascular wall component [19]. Rooney [20] discovered that HA with a molecular weight of 1200–4000 might stimulate fibroblast proliferation and collagen production. Huang Lee [21] conducted an in vitro HA in contracture experiment using a collagen fiber matrix contracture model. It was discovered that when the HA concentration in the matrix reached 1 mg/ml, it greatly inhibited matrix contraction. A high concentration of HA may stimulate the migration of fibroblasts by forming high hydration channels, so impeding the contact between fibroblasts and collagen grids and limiting the contracture of matrix. High-molecular-weight HA may also impede transforming growth factor beta-1 (TGF-β1) and myofibroblast differentiation [22]. Allison [23] revealed that exogenous and endogenous HA affect extracellular matrix differently. Exogenous HA tightens the matrix and compacts collagen. In our animal investigation, the collagen types I and III ratios in the various groups on days 28 and 56 were: TS > PADM > HA1 > HA2 Although collagen types I and III expression increased in HA1 and HA2, their ratios were lower than in PADM. HA2 was below HA1. These data demonstrate HA inference improves skin tension and elasticity. Same outcome in clinical research.

The more exogenous HA, the more collagen types I and III are synthesized. Day 28 HA1/HA2 and PADM differences were bigger than day 56. Unknown whether HA's diminished influence is due to fewer breakdown products or its environment. Whether continued exogenous HA treatment will help is unknown.

## Conclusions

Exogenous HA in ADM composite grafting relieves skin graft contracture and optimizes collagen protein I and III ratio. HA increases the relaxation and stress–strain properties of transplanted skin by promoting vascularization, boosting collagen I and III expression, and reducing the ratio between the two. Exogenous hyaluronic acid mixed with thin skin autograft has greater biomechanical properties and therapeutic effect than acellular dermal matrix alone coupled with thin skin autograft, and the reconstructive result is equivalent to self-thick skin autograft in all indices.

### Supplementary Information

**Additional file 1.** Hypertrophic scar and scar contracture can be seen in this little boy’s left upper extremity. The black circled part was repaired by auto-thin graft, ADM and HA while the other area was repaired by auto-thin graft and ADM. The area with the addition of HA recovered better than the other area.

## Data Availability

The data that support the findings of this study are available from the corresponding author (C.B.G), upon reasonable request.
